# PP81 The Importance Of Networking To Produce Better Health Technology Assessment Evaluations: An HTA Guideline For Orthoses And Prostheses In Brazil

**DOI:** 10.1017/S0266462324002502

**Published:** 2025-01-07

**Authors:** Ketinlly Yasmyne Martins, Rodolfo Castelo Branco, Katia Galdino, Lauriston Medeiros, Eduardo Jorge V Oliveira, Monica Vinhas de Souza

## Abstract

**Introduction:**

Health technology assessment (HTA) studies focused on equipment have particularities. The evaluation of orthoses (ORT) and prostheses (PRT) is even more challenging and tends to present more difficulties when compared to technologies such as medicines. Furthermore, in Brazil there are different manufacturing and dispensing processes for these technologies throughout the country; this heterogeneity could also affect the sustainability of the system itself.

**Objectives:**

(i) evaluate and disseminate the elements necessary to carry out adequate and sustainable assessments of non-implantable ORT and PRT and; (ii) produce a guideline on HTA assessment for non-implantable ORT and PRT.

**Methods:**

Visits were made to ORT and PRT centers throughout Brazil to collect data on the production chain, dispensing, adaptation, and evaluation of the patient’s clinical results. At the same time, systematic literature reviews were carried out on two topics: (i) elements that must be considered in the manufacture, dispensation, and adaptation of non-implantable ORT and PRT; and (ii) HTA guidelines/examples focused on ORT and PRT.

**Results:**

Eight of a total of 12 centers throughout different Brazilian regions were visited. A scoping review was carried out on body-sizing methods for manufacturing non-implantable ORT and PRT, and the differences and particularities of each method. Another systematic review on existing HTA guidelines and the HTA assessment of ORT and PRT is ongoing. The data of the three phases will be analyzed and translated in a Brazilian guideline of HTA for non-implantable ORT and PRT.

**Conclusions:**

With the network of regional and national centers and the sum of different actors (such as government agencies, universities, and health centers), working together for common ground and the sustainability of the health system in complex areas such as HTA of ORT and PRT would be viable. Even with this effort, the work to be done and the challenges are significant.

